# Pooled testing: A tool to increase efficiency of infant HIV diagnosis and virological monitoring

**DOI:** 10.4102/ajlm.v9i2.1035

**Published:** 2020-08-11

**Authors:** Wolfgang Preiser, Gert U. van Zyl

**Affiliations:** 1 Division of Medical Virology, Department of Pathology, Faculty of Medicine and Health Sciences, Stellenbosch University, Cape Town, South Africa; 2 National Health Laboratory Service (NHLS) Tygerberg, Cape Town, South Africa

**Keywords:** HIV, antiretroviral treatment, early infant diagnosis, pooling, pooled testing

## Abstract

**Background:**

Pooled testing, or pooling, has been used for decades to efficiently diagnose relatively rare conditions, such as infection in blood donors. Programmes for the prevention of mother-to-child transmission of HIV and for antiretroviral therapy (ART) are being rolled out in much of Africa and are largely successful. This increases the need for early infant diagnosis (EID) of HIV using qualitative nucleic acid testing and for virological monitoring of patients on ART using viral load testing. While numbers of patients needing testing are increasing, infant HIV infections and ART failures are becoming rarer, opening an opportunity for pooled testing approaches.

**Aim:**

This review highlights the need for universal EID and viral load coverage as well as the challenges faced. We introduce the concept of pooled testing and highlight some important considerations before giving an overview of studies exploring pooled testing for EID and virological monitoring.

**Results:**

For ART monitoring, pooling has been shown to be accurate and efficient; for EID it has not been tried although modelling shows it to be promising. The final part attempts to place pooling into the context of current mother-to-child transmission of HIV and ART programmes and their expected trajectories over the next years.

**Conclusion:**

Several points warrant consideration: pre-selection to exclude samples with an elevated pre-test probability of positivity from pooled testing, the use of dried blood or plasma spots, and choosing a pooling strategy that is both practically feasible and economical. Finally, novel ideas are suggested to make pooling even more attractive.

## Early infant diagnosis of HIV and virological monitoring of patients on antiretroviral therapy – Ongoing challenges for sub-Saharan Africa

The prevention of mother-to-child transmission (PMTCT) of HIV has made enormous progress over the past 15 years, with much-increased coverage and greatly improved programme components. According to estimates for 2017, 80% of HIV-positive pregnant women globally – and as many as 93% in Eastern and Southern Africa – received antiretroviral drugs for PMTCT. While 180 000 children aged up to 14 years were newly infected with HIV globally, 210 000 infant infections were averted due to PMTCT.^1^ Globally, 14.8 million HIV-exposed children remained uninfected.^1^

In South Africa, the national PMTCT programme has followed the World Health Organization Option B+ policy since 2015: Provision of lifetime combination antiretroviral therapy (ART) for all HIV-positive pregnant women, regardless of their immunological or clinical stage.^2^ This has resulted in the reduction of the mother-to-child transmission rate to an estimated 4.6% overall in 2016.^3^

Despite these successes, all HIV-exposed infants continue to need early infant diagnosis (EID). Maternal antibodies persist up to age 18 months. Therefore, EID relies on virological assays that detect either viral nucleic acid or viral proteins at different ages to diagnose cases of prenatal, perinatal and postnatal transmission.^4^ The prompt diagnosis of HIV infection is a prerequisite for starting infected babies on ART early on, thus improving their prognosis.^5^ While infant HIV infection has now become or will soon become a relatively rare event,^6^ the sheer number of exposed infants in need of testing at different time points will continue to pose a major challenge to laboratory systems.

Another area that has seen enormous progress over the past two decades is ART programmes, which have been implemented in much of Africa and have been generally successful. Of the 20 million people estimated to be living with HIV infection in Eastern and Southern Africa in 2017, about 13 million or 66% were on ART, of whom more than 10 million were estimated to have suppressed viral load (VL).^1^ For South Africa, according to 2017 estimates, 90% of HIV-positive individuals know their status; 68% of these are on ART and 78% of those on ART achieve viral suppression.^1^

Previously regarded as optional,^7^ routine virological monitoring of patients on ART by HIV VL testing to detect ART failure allows optimal patient management. Addressing either adherence or presumed drug resistance (when there is no response to adherence interventions) secures long-term viability of ART programmes by limiting emergence and transmission of antiretroviral drug resistance.^8,9^ Optimal virological suppression is essential to prevent onward transmission; therefore, achieving an undetectable VL in 90% of those on ART has been included as the last component of the Joint United Nations Programme on HIV/AIDS (UNAIDS) 90-90-90 targets to help end the HIV/AIDS epidemic.^10^ The need for virological monitoring is increasingly reflected in ART guidelines.^4^

Yet significant barriers remain: currently available VL tests are expensive, technically demanding and thus not widely available in many resource-limited settings (RLS).^11^ While point-of-care VL tests may be an ideal solution and several systems are approaching clinical usability, their relatively high cost and other challenges remain unresolved.^12^ A recent forecast study underlines the need for a substantial rollout of both EID and VL testing.^13^

## The concept of pooled testing (‘pooling’) – Some important considerations

Pooled testing or ‘pooling’, also known as ‘group testing’, refers to mixing several samples together prior to performing a laboratory test. Individual samples contained in a negative pool can be classified as negative without further testing, whereas samples contained in pools testing positive need to be re-tested individually.

While pooling may seem rather crude and does not apply to measurements of analytes that are present in both physiological and pathological states, albeit, at different concentrations, pooling has a long tradition for infectious disease markers, for example for the screening of blood donations.^14^ Here the physiological state is ‘negative’ (which means the laboratory marker, for example specific antibodies or pathogen-specific nucleic acid sequences, are undetectable), thus, the prevalence of positive samples among donors is low, thanks to the application of stringent donor selection criteria. Used for antibody-based screening (e.g. syphilis) for decades, pooled testing was later adopted for nucleic acid testing (NAT), too.^15^

The advantage of pooling lies in its ability to save on the number of tests needed and, thus, costs. As long as the condition to be detected occurs at low prevalence, most pools will test as negative. Then, all samples contained in a negative pool can have a negative result reported while having used just one test. If a pool tests positive, it needs to be determined which of the constituent samples is responsible. This is achieved by re-testing the constituent samples individually (a process termed ‘deconvolution’ or ‘resolution’) which thus negates any saving for the samples concerned, in that the constituent samples together will, in the end, have used a number of tests equal to their number plus one (the test used for the pool).

Alternative strategies have been proposed that are more efficient than using straightforward pooling and deconvolution by re-testing constituent samples simultaneously. In two-dimensional or multidimensional matrix approaches, each sample is contained in a unique combination of different pools; the combination of pools that test positive resolves the sample responsible without re-testing.^16^ Multistage or pyramid-type pooling strategies may likewise be more efficient than simple pooling.^17^ When a pooling approach is used for quantitative tests, a strategy of individually re-testing the constituent samples one after the other may be designed; when an individual sample’s quantitative test result can explain the quantitative result for the pool, the remaining samples may be regarded as negative.^18^ Such an approach is, however, not without challenges, especially given the test-to-test variation of quantitative results.

The main factors determining whether pooling may be advantageous are the prevalence of the condition to be tested for, and the pool size. Pooling is predicated on a relatively low prevalence condition being sought. If a condition is highly prevalent, pooling will not save tests, due to frequent deconvolution and individual re-testing. The larger the pool size (i.e. the number of samples mixed together to form one pooled specimen for testing), the greater the potential saving. However, pool size is not only limited by practical considerations – test volumes and dilution factors, the latter linked to test sensitivity; but by the tested condition’s prevalence – a low prevalence ensures that a substantial proportion of test pools are negative. A simple example illustrates this: assuming a prevalence of 20%, a pool size of five specimens will yield almost no savings and a pool size of four, only marginal savings.^19^

By excluding patient samples with a high probability of positivity from pooling, the prevalence of the condition tested in the pooled samples is reduced invariably improving pooling efficiency.^20^ Examples of such patients are children whose mothers did not receive PMTCT, those who failed ART previously or are clinically unwell, and those sent for confirmation of a previous positive result.

Several aspects need to be taken into account when considering pooled testing. The first aspect is the test sensitivity. Pooled test sensitivity is often somewhat lower than the sensitivity of individual tests because of the required dilution of pooled specimens. However, this lowered may not be clinically relevant and counteracted to some degree by modifying the testing process (e.g. substituting some of the diluent or sample buffer with specimen). The sensitivity of pooled testing and any measures to improve it has to be considered and validated carefully before using pooling for diagnostic purposes. Currently, available commercial VL assays have lower limits of detection below 50 copies/mL. This redundant sensitivity can be exploited by testing pools consisting of several mixed samples, if the World Health Organization’s recommended threshold is used for clinical decision-making.

Sample throughput is another important consideration. There needs to be a balance between volumes of samples (requests) received per day and the pool sizes (to allow for efficient pooling) without increasing test turn-around time while waiting for enough specimens to be received to allow for pooling.

Finally, the processes of pooling and deconvolution (of positive pools) add to the operator workload. On the other hand, operator workload is reduced through efficient pooling, as fewer tests need to be performed. However, performing pooling and deconvolution may also introduce clerical errors; the approach must therefore be designed well and conducted carefully. These factors need to be factored into calculations of pooling efficiency and assessing practicability.

The interaction between the above-listed factors is rather complex. It is highly unlikely that anyone or a limited choice of pooling solutions, would be suitable for different scenarios.

### Pooled testing for early infant diagnosis of HIV

The earliest possible diagnosis of HIV infection in HIV-exposed infants is a prerequisite for early initiation of ART, which markedly improves the prognosis of infants with HIV infection.^5^ This requires testing uncoagulated whole blood or dried blood spot (DBS) samples for viral genome (proviral DNA, viral RNA, or both) by means of polymerase chain reaction (PCR) or similar genome amplification technologies.^4^ PCR is technically demanding and costly, limiting access to it in many RLS. DBS are widely used in RLS, as they are easier to obtain, to store and to transport while performing generally well.^21^ Recommendations about the optimal time points for EID testing are evolving, due to programmatic improvements and new scientific findings. The optimum would be three EID tests for each exposed infant: the first at or soon after birth (to detect infection acquired in utero), the second at around 6–10 weeks of age (to detect perinatal transmission; the preferred timing depends on the duration of postnatal post-exposure prophylaxis), and a third one 6 weeks after cessation of breastfeeding (to detect postnatal transmission, which would usually be via breast milk).^2^

Not only is EID coverage across most of sub-Saharan Africa poor and the results commonly delayed,^1^ performing three tests for each exposed infant also places an enormous burden on health and laboratory services. Measures to increase EID capacity and reduce costs are therefore sorely needed. Point-of-care assays are increasingly being made available and have been piloted in several studies, but several drawbacks remain unresolved: their relatively high complexity requires trained staff; their relatively low throughput requires multiple instruments at busy clinics; and their relatively high cost which is and will likely remain higher than testing at centralised laboratories.^22^

Interestingly, there does not seem to be much interest in pooled testing for EID. The first and so far only known study on this topic, published in February 2019, determined the clinical accuracy of pooled testing of infant DBS and developed a model to simulate the saving of reagent and consumable costs based on real data from a South African public health laboratory.^23^

The study found a high sensitivity (98.8%) for pooled testing. The one observed false-positive pool test result was confirmed through re-testing of the constituent patient samples. The savings of laboratory costs – 64% based on 2015 prices – were substantial. The article concludes that pooling EID PCR tests retains accuracy while substantially reducing costs, provided a laboratory receives sufficient numbers of samples and has a low to moderate infant HIV prevalence.

The authors have developed and made available publicly an R-based web tool to assess the cost-efficiency of pooled PCR testing for infant HIV diagnosis for varying parameters (https://carivs.shinyapps.io/Calculator). Users will enter the average number of samples tested per day, the estimated positivity rate (maximum allowed is 10% as above that pooling would not be viable), the sample type (DBS or whole blood), the cost of reagents (if unknown, the model provides an estimated percentage saving) and the minimum number of samples required to run a batch for pooling and for individual testing. The model will then do a calculation, based on the above, and report the optimal pool size, the cost saving, and the reduction in daily batch runs both as a sentence (e.g. Based on your input values, at the optimal pool size of 5, you will save 62% of costs and daily batched runs will decrease from 2 to 1) and graphically as a three-dimensional plot with axes for positivity (%), pool size and cost as a percentage of cost for testing individual samples.

### Pooled testing for virological monitoring

The sustained suppression of HIV replication in patients on ART is the goal of HIV treatment. Halting viral replication provides individual benefit as it allows immune reconstitution and reduces inflammation, thereby lowering the risk for HIV-associated infectious and non-communicable complications. Moreover, on a population level, sustained virological suppression renders treated patients virtually non-infectious and thus prevents onward transmission.^24^

Conversely, failing to achieve virological suppression puts the patient and the population at risk; this is not only through HIV disease and the transmission of HIV infection, but also through the emergence of antiretroviral drug-resistant HIV strains which may jeopardise future ART options for the individual and, through the transmission of resistant virus strains could put the whole ART programme at risk of failure.^25^ Monitoring of patients on ART by means of virological assays is therefore of paramount importance and after years of advocacy, this is now widely acknowledged and reflected in the third ‘90’ of the UNAIDS’s 90-90-90 goals.^26,10^

In 2017, the proportion of people of all age groups on ART who achieved virological suppression was estimated at 81% globally, at 79% for Eastern and Southern Africa and at 73% for West and Central Africa. For Botswana, the estimate is > 95% and for South Africa, 78%. Virological non-suppression appears to be increasingly rare in many settings and may have become a low prevalence condition in some places already.^1^

Virological monitoring is usually done through HIV VL testing, that is, using assays that quantify the concentration of HIV RNA in patient plasma. A VL result of less than 50 copies of HIV RNA per millilitre of plasma is usually regarded as full suppression, which is the aim of ART in industrialised countries. In RLS, VL of 1000 copies/mL in patients on ART for at least 6 months or a VL above 1000 copies/mL in patients after two consecutive measurements 3 months apart with adherence support defines virological failure (VF) and should trigger clinical action.^4^

The rationale for using 1000 copies/mL as the widely accepted public health threshold is twofold: below this level, there is a limited risk of both disease progression and HIV transmission.^4^ Additionally, it avoids the irrelevant detection of viral ‘blips’, short episodes of low-level viraemia that may occur during effective ART and are not associated with an increased risk of ART failure.^27^

Smith et al.^28^ was the first study to report the use of pooled VL testing to identify ART failure. It was conducted on a small number of ART patient samples in the United States and compared two different pooling strategies, namely mini pools of five samples each and 10 × 10 matrix pools, with individual VL testing as the gold standard. In a matrix pooling approach, each sample is contained in more than one pool and the combination of pools testing positive allows direct identification of the sample responsible in most instances when the prevalence of failure is low, making deconvolution unnecessary. Even though 23% of samples had a VL of > 50 copies/mL, both pooling strategies combined with a search and retest algorithm, led to savings of > 50% compared with individual testing, with only a minimal decrease in accuracy.

A subsequent modelling study assessed various pooling strategies for monitoring ART patients with VF prevalences between 1% and 25% in terms of efficiency and accuracy.^19^ It also compared three different pooling approaches – mini pools, mini pools with algorithm-based deconvolution, and matrix pooling to individual testing. Using the quantitative information, that is, VL measurements available, this study showed pooling efficiency at higher prevalences than for which a purely binary approach (VL either detectable or undetectable) would have been efficient. However, such relatively complicated approaches may not prove feasible in many RLS.

A third study, conducted in South Africa, investigated three aspects. Firstly, whether the use of basic data recorded on routine laboratory request forms would allow selection of ART patients with a low probability of ART failure for pooled testing. Secondly, whether mini pool or matrix pooling strategies were more suitable, comparing them against individual VL testing in a laboratory study. Thirdly, the use of DBS and dried plasma spots instead of liquid plasma samples for preparing mini pools.^20^

In the retrospective cohort studied, the VF rate was 14.2%.^20^ After applying age > 15 years and being on a first-line ART regimen as selection criteria (information easily obtained from test request forms), this dropped to 8.7% making pooling of such pre-selected samples more feasible. Four hundred plasma specimens were tested in 80 mini pools of five each, and 300 of these samples also with a 10 × 10 matrix strategy. Pooled testing reduced the number of tests needed by 30.5% – 60.0%. Even though the matrix pooling strategy was more efficient than mini pooling, the latter was deemed overall more suitable as it proved much easier to perform, taking less operator time and requiring less expertise for constitution and deconvolution of pools. In addition, waiting for 100 samples to be available for testing before a 10 × 10 matrix can be constructed may increase turn-around times in many laboratories; this is generally not a problem with mini pools of five samples. The study found a poorer specificity and efficiency of pooled DBS testing. Factors such as the contribution of cell-associated HIV RNA and the variable plasma fraction (depending on patients’ haematocrit values) could cause falsely high VL results, while the presence of haemoglobin and other inhibitory substances in DBS could lead to falsely low results. Pooled dried plasma spot testing, however, showed less variability and excellent accuracy and an efficiency of 60% for a pool threshold of 200 HIV RNA copies/mL.

A large field study in Malawi subsequently addressed two issues relevant to RLS: a higher VF rate and the problems inherent in testing plasma samples.^29^ Testing 350 patient samples, the study found that pooled testing of plasma samples had excellent sensitivity (96.4%) and reduced the number of tests required by 44.3%. Using DBS, prepared either directly by finger prick (which would be the preferred option in real-life use of pooled testing in RLS) or from venous blood, sensitivity was 78.6% and 89.3%, saving 28.6% – 32.9% of tests. It concluded that when using the NucliSENS assay – which does not amplify the cell-associated HIV DNA contained in DBS, in contrast to most other HIV VL assays – testing DBS is a feasible option.

Pooling for virological monitoring of ART patients has been assessed in other settings: those with a low prevalence of VF such as South Korea^30^ and in RLS with typically much higher failure rates such as Mexico,^31^ Kenya^32^ and Mozambique.^33^ Even under RLS conditions pooled testing was generally efficient with acceptable accuracy, although not as efficient as in settings with low failure rates. However, El Bouzidi et al.^34^ reported that testing in mini pools of four samples could not reliably identify VF in a London, United Kingdom, ART population, due to insufficient diagnostic sensitivity. While almost one in three failing patients was missed, the VL values of failing patients were low and mostly irrelevant for RLS.

## Perspectives and outlook

Pooled testing has a long history of successful use in the field of infectious disease testing, the most notable application being for screening blood donors both by serology and since the 1990s by NAT.^15^ More recently, a multitude of studies have investigated pooled NAT as a tool to detect early HIV infection that would be missed by routine HIV screening tests. In Africa, Karim et al.^35^ were the first to propose such a strategy; yet even in a high risk setting such as described by Gous et al.,^36^ pooled NAT would still be relatively expensive per additional case detected, as very few patients will be in the NAT-positive antibody-negative stage of recent infection.^37^ More recently, a study by Dowling et al.^38^ demonstrated that DBS can be used for this purpose, which would facilitate its wide implementation in RLS.

With ongoing major advances in both EID and ART programmes, it is surprising that the use of pooled testing has hardly been explored for the purposes of EID, while there is ample literature on pooled testing for monitoring patients on ART in a variety of settings.

The need for EID testing will remain unchanged even as mother-to-child transmission rates are in decline. Point-of-care testing might appear as an ideal solution for a variety of reasons,^39^ yet it is questionable whether it will be the most affordable solution against the background of an enormous, only partially met need. Pooled EID testing has been modelled to be efficient,^23^ and infant HIV infection is becoming less prevalent in most places. However, ongoing vigilance is required as the evolving nature of the PMTCT programme makes establishing a definite diagnosis increasingly challenging.^40,41^

The need for ART monitoring will increase in the foreseeable future. In principle, when the prevalence of VF is low, pooled testing is very efficient, saves costs and may even reduce the average turn-around time (time from obtaining sample to receipt of test result). It is expected that with the rollout of newer therapy regimens the prevalence of VF may drop further, to below 7%.^42^ This might cause pressure to decrease the frequency of VL testing further, when in actual fact failure needs to be identified more efficiently. The performance characteristics of modern laboratory-based VL assays are redundant for widely accepted VL thresholds and thus provide comfortable room for pooling; highly sensitive assays offer leeway when a threshold of 1000 copies/mL is used to define ART failure. Pooling would potentially increase throughput and reduce turn-around time.

Pooled HIV VL testing has the potential to reduce the reagent cost of VL testing. Early initiation of ART and the use of more tolerable high genetic barrier regimens for first-line ART have the potential to further drive down the prevalence of VF.^42^ Pooled VL testing is therefore likely to become even more efficient in the near future. Nevertheless, large scale implementation of pooled testing requires overcoming several hurdles. The first is susceptibility to human error, when pooling and deconvolution are performed manually. The second is that pooling and deconvolution add to hands-on time and therefore increase labour costs. The third is that the efficiency of pooled testing is highly susceptible to test failure.

### Important considerations

As regards pooling for EID and monitoring ART, a number of pivotal points are emerging from the published studies, and these issues need to be taken into account when considering the introduction of pooled testing.

Firstly, to ensure the highest possible efficiency of pooling, a pre-selection step should be considered to identify samples from patients with a high pre-test probability of VF (i.e. HIV-positive patients). This could be achieved by interrogating the information contained on most test request forms or the information contained in the laboratory information system, where possible. The purpose of this would be to exclude ‘high risk’ specimens from being pooled lest they ‘poison’ pools.

Secondly, to maximise the usefulness of pooled testing in RLS, the use of DBS or dried plasma spot would be ideal. Once dried onto filter paper, such samples are non-infectious and stable for prolonged periods, as long as they are not subjected to extreme temperatures and excessive humidity; they can therefore be stored and transported even over long distances easily and cheaply.^43^ Dried blood spots are relatively easy to obtain using finger (or toe or heel, in infants) pricks and are already widely used for EID in RLS. For EID, their sensitivity may need consideration as the proportion of infected infants with low VL values increases.^44^ Regarding VL testing, DBS have certain intrinsic disadvantages that are unlikely to be overcame, such as the detection of intracellular viral genomes. On the other hand, dried plasma spots negate part of the attractiveness of dried samples as they require expert sample handling and manipulation at the clinical site. This expertise is often lacking in many peripheral clinics and also increases operator time, which in turn increases the risk of sample mix-ups, mislabelling, and the like. How much solution simple-to-use plasma separation devices will offer for this issue remains to be ascertained.^45^

Thirdly, regarding the preferable pooling strategy, it appears that in RLS straightforward mini pools and parallel deconvolution probably offer the best efficiency with ease of handling. In many RLS it will be necessary to avoid placing too much extra strain on scarce laboratory staff in terms of work time and effort. A misconceived, over-ambitious approach may lead to an increase in clerical errors and other problems that could easily negate the efficiency gained through pooling.

### Limitations

Apart from the factors outlined above, a clear limitation of pooling is that it needs to be done in a centralised laboratory in order to have access to preferably automated equipment and skilled staff to operate it and to assemble large enough numbers of specimens for testing. This necessitates sample transport, which is often challenging and increases turn-around times. Point-of-care testing obviously has a number of advantages over any centralised testing, including pooled testing. However, it also poses a number of challenges such as price, operator training and supervision, quality control, etc., which still have to be resolved. Ultimately, both will have a role to play: point-of-care testing for remote rural clinics (to avoid expensive shipping and lengthy delays) and pooling for urban areas and clinics with good transport links to major laboratories.

### Suggestions for future research

Two opportunities exist that could make pooling even more attractive for RLS. The first can be summarised by the question whether the ‘load’ in ‘viral load’ is actually needed. Using a qualitative HIV PCR assay calibrated to detect down to approximately 1000 copies/mL per constituent sample and which generates a product covering most of the HIV *pol* gene for Sanger sequencing, pooled testing could inform about VF (albeit without assigning a VL value) and the presence of the most relevant drug resistance mutations. This unconventional approach seems to work well in both developed countries and in RLS,^46,47,48^ while addressing the urgent and largely unmet need for resistance testing in Africa.^49^

The second opportunity addresses a major challenge for most African laboratories: the scarcity of well-qualified and trained staff. Pooled testing has one principal disadvantage: it requires additional handling and, thus, increases hands-on time for qualified staff. This is necessary to perform the additional steps involved, namely the preparation of the pools and then the deconvolution of positive pools, involving retrieval of the samples that comprised a positive pool and re-testing them. Unfortunately, both of these tasks are prone to human errors, which can cause sample mix-ups and other mistakes.

Automated pooling may help to overcome the important implementation hurdle posed by the shortage of trained laboratory staff. A pre-analytic sample robot could conduct the actual pooling process, then store samples (preferably cooled) until the pooled test result is available and lastly retrieve samples from positive pools for deconvolution. Additionally, such a system could be integrated with the laboratory information system and also pre-screen patient samples with a low probability for failure, before preparing the pools educing the need for deconvolution.

To conclude, pooling offers attractive prospects for African countries, not just for monitoring ART but also for conducting EID testing. The time has come to conduct large-scale pilot studies, first on a limited scale, in parallel to individual testing to demonstrate non-inferiority of pooled testing in everyday use and subsequently in settings with suboptimal EID availability or which do not currently offer routine regular virological monitoring.
